# A Case Report: Ertapenem-Induced Encephalopathy

**DOI:** 10.7759/cureus.96107

**Published:** 2025-11-04

**Authors:** Russell Boonchai Lim, Baoxian Huang, Aaron Zijie Meng

**Affiliations:** 1 Psychiatry, Ministry of Health Holdings, Singapore, SGP; 2 Psychiatry, Ng Teng Fong General Hospital, Singapore, SGP

**Keywords:** antibiotics induced encephalopathy, antibiotics induced psychiatric symptoms, antibiotics induced psychotic symptoms, antibiotics side-effect, consultation liaison psychiatry, drug-induced encephalopathy, encephalopathy, ertapenem induced encephalopathy, ertapenem side-effect, neurotoxicity

## Abstract

We present a 46-year-old patient who developed acute psychotic and neurological symptoms associated with the use of intravenous ertapenem, a carbapenem antibiotic. He was hospitalized following a road traffic accident where he sustained an open fracture over the right lower limb requiring intravenous antibiotics. He started to exhibit hallucinations, odd beliefs and twitching movements two weeks after the initiation of ertapenem. Other organic causes for this acute change in behaviour were ruled out. His symptoms continued despite the use of antipsychotics but quickly resolved within three days of the withdrawal of ertapenem.

Antibiotic-induced encephalopathy, which can manifest as neurological or psychiatric symptoms, is uncommon but clinically significant. Among these, carbapenems have been occasionally associated with encephalopathy through GABAergic inhibition. This case highlights the importance of recognizing ertapenem-induced neuropsychiatric complications even in patients without conventional risk factors, underscoring the need for vigilance when evaluating acute behavioural changes during antibiotic therapy.

## Introduction

Routinely used medications have been known to cause psychiatric adverse effects. Patients may present with symptoms of psychosis, mania, depression, or anxiety, with accompanying agitation, aggression, and even suicidal or homicidal behaviours [1].

These psychiatric symptoms may result in adverse clinical outcomes, including an increased rate of falls, mortality, a lack of cooperation with treatment, increased length of hospitalization, and cost of care [1]. The diagnosis of medication-induced psychiatric disorders may be complicated by a prior psychiatric history, substance use history, lack of corroborative history, as well as active clinical conditions for which the patient is undergoing treatment.

Common medication classes known to precipitate the onset of psychiatric symptoms include steroids [2], anti-cholinergics [3], opioids [4], and sedatives [5].

Nearly all antibiotic classes have been associated with rare and reversible, though potentially severe psychiatric effects [6]. It is hence important for mental health professionals in the Consultant Liaison setting to recognize antibiotics as a possible cause for the presentation of psychiatric symptoms.

Fluoroquinolones via gamma-aminobutyric acid (GABA) antagonism and N-methyl-D-aspartic acid (NMDA) activity may result in restlessness, mania, anxiety, psychosis, delirium, and seizures [7]. Metronidazole via GABA antagonism may result in seizures, encephalopathy, and psychosis [8]. Through varied mechanisms, almost all classes of antibiotic medications may result in the presentation of psychiatric symptoms.

Carbapenems are broad-spectrum antibiotics used in the context of serious bacterial infections. A rare adverse effect of carbapenems includes carbapenem-induced encephalopathy. The beta-lactam ring is structurally similar to the GABA neurotransmitter [9]. Carbapenems may hence act on the central nervous system directly via GABA-A antagonism. This results in dose-dependent adverse effects of seizures, hallucinations, delirium, and psychosis [10,11]. Risk factors for such psychiatric adverse effects include alcohol use, renal failure, advanced age, chronic benzodiazepine use, vigabatrine use, and valproic acid derivative use.

This case report aims to present the signs and symptoms in a probable ertapenem-induced encephalopathy in the inpatient setting.

## Case presentation

A 46-year-old Chinese man with a known history of glucose-6-phosphate dehydrogenase (G6PD) deficiency presented to our Emergency Department in July 2023 following a road-traffic accident. He had skidded off his motorcycle at around 70 kilometres per hour and landed on his right side. He complained of right lower limb pain but denied any head injury or loss of consciousness. He had consumed seven glasses of wine prior to the incident.

Premorbidly, this patient did not have any known past psychiatric or illicit drug use history.

On physical examination, the patient had extensive friction burns on the right anterior abdominal wall, left forearm abrasions, right-sided shin and knee abrasions, and an open wound on the medial aspect of the right ankle with active bleeding and deformity. In terms of consciousness level, his Glasgow Coma Scale (GCS) score was noted to be 15, and he was alert and oriented with no signs of head injury. An Abbreviated Mental Test (AMT) was performed with the patient scoring 10/10.

A bedside ultrasonographic assessment did not show the presence of abdominal free fluid, pericardial effusion, nor any signs of pulmonary pathology. A blood toxicology screen revealed ethanol at 195mg/100ml. There was also a positive finding of piroxicam and tadalafil in the blood toxicology report which could not be further verified as the patient denied a history of taking these substances. 

X-ray imaging revealed fractures of the right second to fourth metatarsal necks, and a fracture of the right distal fibula with a fracture of the posterior malleolus with disruption of the ankle mortise. 

He was reviewed by the Orthopaedics team and underwent surgery for a right ankle open bimalleolar fracture dislocation. 

In the ward, he was given paracetamol, tramadol, and etoricoxib as analgesia. He was also started on intravenous cefazolin and given a tetanus toxoid injection.

During the initial phase of his admission, he had to undergo multiple surgical procedures, including open reduction and external fixation of the right ankle, Lisfranc fracture fusion, midfoot stabilization and wound debridement. During the eighth day of his admission, his treatment was complicated by a necrotizing *Escherichia coli* and *Bacteroides fragilis* infection over his surgical site, which required central venous line access and escalation of antibiotics. In consultation with a microbiologist, his antibiotic regimen was changed to intravenous cefepime and metronidazole for a day. For local infection control, antibiotic beads were inserted into his wound with simultaneous negative pressure wound therapy. The implants were removed and an external fixator was applied to his right ankle. Antibiotic therapy was subsequently switched to intravenous ertapenem and metronidazole following sensitivity results, with an aim for eight weeks of continued therapy.

The patient clinically improved and was able to undergo wound coverage via a latissimus dorsi flap and left thigh skin graft. He remained on culture-guided antibiotics and an external fixator at this time. He did not exhibit any behavioural or psychiatric symptoms and was deemed competent to provide informed consent for his various surgeries.

The acute change in behaviour occurred four weeks into the admission, where he became agitated and frustrated overnight and had disrupted sleep. He became uncooperative and was observed to be talking and laughing to himself. He reported auditory hallucinations and appeared intermittently confused and disoriented. These symptoms interfered with clinical care and resulted in non-adherence to his non-weight-bearing instructions.

His psychiatric symptoms evolved over the next few days, where he reported hallucinations of other modalities, including being able to see his “friends” around him, as well as "puppies" biting at his leg. He was also experiencing constant “tapping” sensations over his hands and legs. He also reported odd, grandiose beliefs of being able to teleport downstairs and was often laughing and shouting. 

Corroborative history from the patient’s relatives revealed that the patient did not have a history of recreational drug use nor chronic alcohol use, and had no previous psychiatric issues. He was known to be pleasant in demeanour and had normal intellectual functioning at baseline. This was also consistent with clinical observations of the patient during the initial phase of his admission to the hospital. 

Mental state examination revealed a middle-aged man in hospital garb gazing around the room with poor eye contact. He was tremulous, restless, and fidgeting and was slightly agitated. He was tangential at times and spoke in short rapid phrases. He later revealed that he was also experiencing visual hallucinations as described above.

A brief neurological examination revealed generalized hyper-reflexia on all four limbs. His lower limbs also displayed three beats of clonus. He also displayed a general jitteriness and occasional jerky movements of his upper limbs.

Over the next two days, he underwent thorough investigations to rule out other organic causes for this acute change of behaviour. 

**Figure 1:**
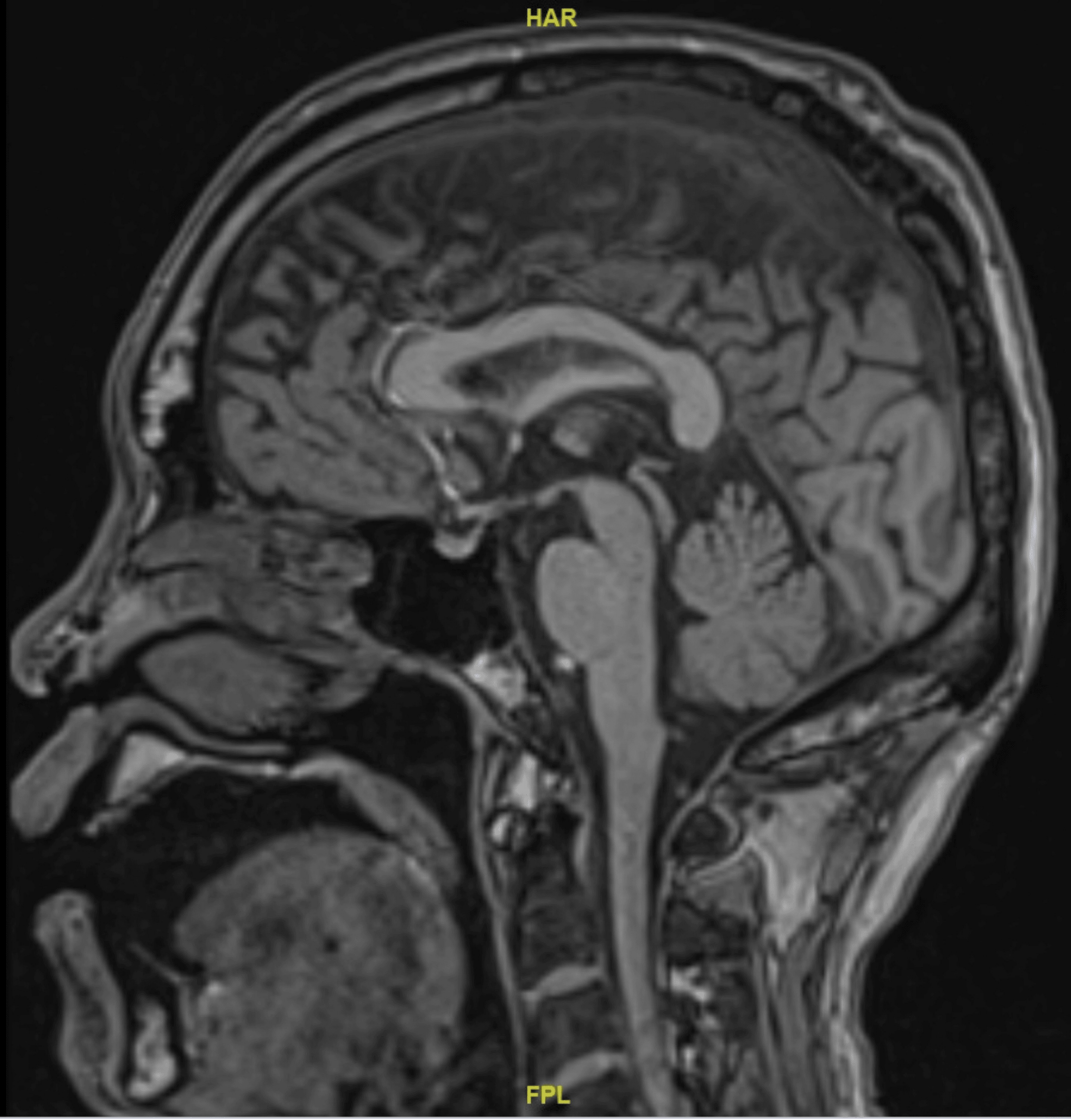
Non-contrast magnetic resonance imaging of the patient's brain, mid sagittal section Permission to use the above brain image for the purpose of this case report was obtained from Ng Teng Fong General Hospital.

**Figure 2:**
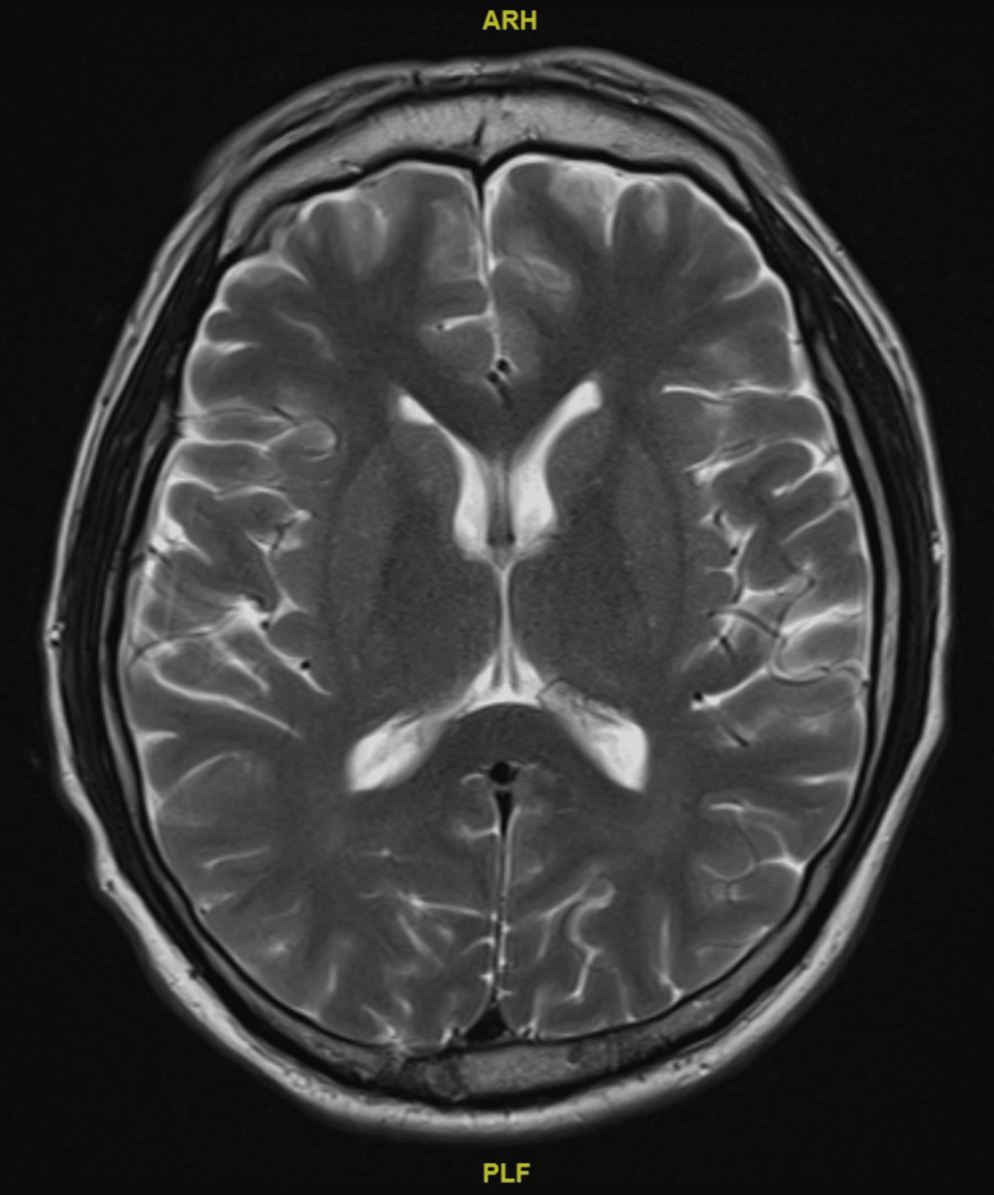
Non-contrast magnetic resonance imaging of the patient's brain, axial section Permission to use the above brain image for the purpose of this case report was obtained from Ng Teng Fong General Hospital.

**Figure 3:**
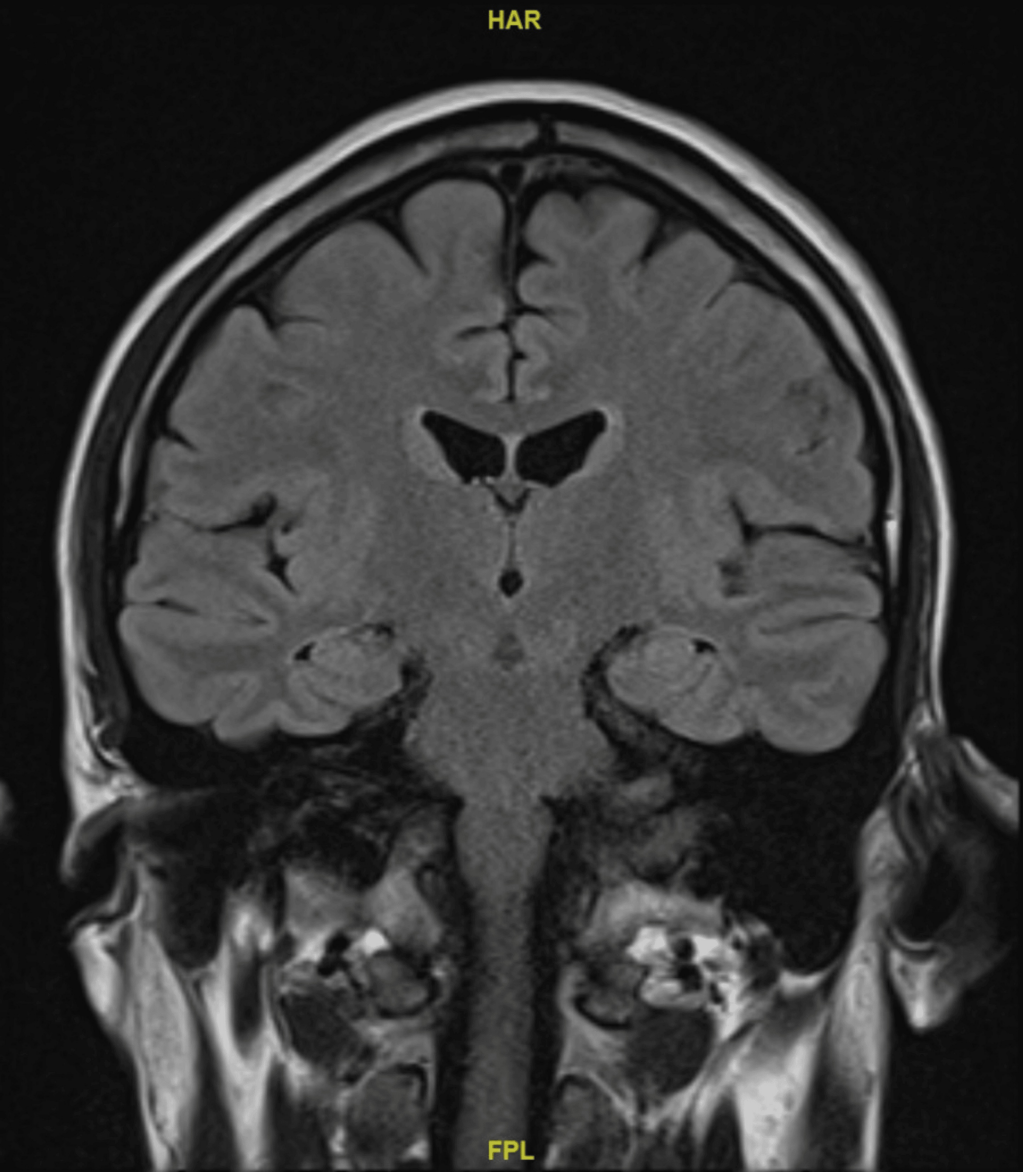
Non-contrast magnetic resonance imaging of the patient's brain, coronal section Permission to use the above brain image for the purpose of this case report was obtained from Ng Teng Fong General Hospital.

A contrasted magnetic resonance imaging of the brain did not detect any significant brain abnormality (refer to Figures 1, 2, 3).

There was also no dyselectrolytemia, liver, or renal impairment, and inflammatory markers were not suggestive of any worsening infection. Serum albumin, however, was noted to be mildly low at 30g/L. Total white cell count was mildly elevated since the start of infection, but were downtrending after the use of culture-directed antibiotics (refer to Table 1). He did not undergo EEG and a subsequent lumbar puncture was held off after his symptoms improved following changes to his medications. 

**Table 1:** Blood tests performed as part the evaluation for his acute change in behaviour MCV: mean corpuscular volume, MCH: mean corpuscular hemoglobin, MCHC: mean corpuscular hemoglobin concentration, MPV: mean platelet volume, eGFR: estimated glomerular filtration rate, CKD-EPI: Chronic Kidney Disease Epidemiology, ALP: alkaline phosphatase, TSH: thyroid-stimulating hormon

	Latest Reference Range & Units	Result
White Blood Cell	4.30 - 10.40 X10ˆ9/L	14.90 (H)
Haemoglobin	13.1 - 16.8 g/dL	9.4 (L)
Haematocrit	40.3 - 50.0 %	29.4 (L)
Red Blood Cells	4.50 - 5.75 X10ˆ12/L	3.30 (L)
MCV	80.6 - 96.1 fL	89.1
MCH	26.1 - 32.1 pg	28.5
MCHC	30.8 - 34.9 g/dL	32.0
RDW	11.5 - 14.5 %	14.7 (H)
Platelets	150 - 410 x10ˆ9/L	578 (H)
MPV	8.7 - 12.1 fL	10.7
Neutrophils %	%	50.1
Neutrophils Absolute	1.90 - 6.53 x10ˆ9/L	7.46 (H)
Lymphocytes %	%	30.9
Lymphocytes Absolute	1.21 - 3.56 x10ˆ9/L	4.61 (H)
Monocytes %	%	8.4
Monocytes Absolute	0.23 - 0.82 x10ˆ9/L	1.25 (H)
Eosinophils %	%	9.5
Eosinophils Absolute	0.05 - 0.50 x10ˆ9/L	1.42 (H)
Basophils %	%	1.1
Basophils Absolute	0.02 - 0.09 x10ˆ9/L	0.16 (H)
High-Sensitive C-Reactive Protein	<=5.0 mg/L	5.1 (H)
Urea, Serum	3.2 - 7.4 mmol/L	6.5
Creatinine, Serum	60 - 110 umol/L	64
eGFR (CKD-EPI)	>90 mL/min/1.73m2	112
Bicarbonate, Serum	22 - 32 mmol/L	21 (L)
Sodium, Serum	135 - 145 mmol/L	139
Potassium, Serum	3.5 - 5.2 mmol/L	4.3
Magnesium, Serum	0.70 - 1.10 mmol/L	0.77
Calcium, Corrected	2.10 - 2.55 mmol/L	2.13
Calcium, Serum	2.10 - 2.60 mmol/L	1.93 (L)
Phosphate, Serum	0.75 - 1.50 mmol/L	1.10
Chloride, Serum	95 - 110 mmol/L	110
Anion Gap	8.0 - 16.0 mmol/L	12.3
Total Protein, Serum	60 - 80 g/L	58 (L)
Albumin, Serum	35 - 52 g/L 35 - 52 g/L	30 (L) 30 (L)
Bilirubin, Total	1 - 20 umol/L	4
Bilirubin, Direct	<=9 umol/L	2
Aspartate Transaminase	6 - 35 U/L	20
Alanine Transaminase	6 - 40 U/L	17
ALP	30 - 110 U/L	123 (H)
Vitamin B12	138 - 652 pmol/L	261
Folate	7.0 - 46.4 nmol/L	10.1
Free Thyroxine (FT4)	9.0 - 19.1 pmol/L	13.8
TSH	0.35 - 4.94 mIU/L	1.73

His psychotic symptoms were treated with olanzapine, which was eventually uptitrated to up to 10mg a day. Despite pharmacotherapy, he continued to exhibit symptoms.

As most of the investigation results were unremarkable, his medications were also reviewed and streamlined to minimize contribution to his change in mental state. Metronidazole and tramadol were stopped, and ertapenem was converted to piperacillin-tazobactam (refer to Table 2).

**Table 2:** Antibiogram of this patient "X" represents the week of the patient's hospitalisation where he was exposed to the specific antibiotic

Antibiotic/duration(week)	1	2	3	4	5	6
Cefepime	X	-	-	-	-	-
Meropenem	X	-	-	-	-	-
Metronidazole	X	X	X	X	X	-
Ertapenem	X	X	X	X	X	-
Piperacillin-tazobactam	-	-	-	-	X	X

His behaviour and psychiatric symptoms were monitored closely throughout the day and with each medication change. What was most notable was a rapid reduction of psychiatric symptoms within three days after his antibiotics were switched from ertapenem to piperacillin-tazobactam. 

Within three days following the change in antibiotics, his mental state and behaviours improved and returned to baseline, allowing the removal of restraints and a resumption in physical therapy. The patient could not recall his earlier episodes of agitation, and described feeling like his normal self. His mood state was normalized and he no longer experienced perceptual abnormalities. Neurological symptoms, such as twitching and hypereflexia, were also resolved.

He was eventually weaned off olanzapine and there were no recurrences of abnormal behaviour for the rest of his hospital stay and during follow-up appointments.

## Discussion

Our case demonstrates ertapenem-induced encephalopathy presenting predominantly with psychiatric manifestations-hallucinations, delusions, disorganized behaviour, and agitation-alongside neurological features of myoclonic jerks, tremors and hyperreflexia. The symptoms occurred around day 14 of administration of ertapenem and resolved within three days of withdrawal of ertapenem. The Naranjo scale [12] was applied in our case and achieved a score of 6, supporting the conclusion of a probable adverse drug reaction (refer to Table 3). Differential diagnoses, which include other potential underlying organic causes of delirium, seizures and other central nervous system conditions, were systematically investigated and ruled out, with notable exceptions of lumbar puncture and EEG investigations which were both planned but later cancelled as they were no longer indicated due to the rapid resolution of the patient’s symptoms.

**Table 3:** Naranjo Scale (Adverse Drug Reaction Probability Scale) The scale is as follows: >9 definite, 5–8 probable, 1–4 possible, <0 doubtful [12].

Question	Yes	No	Do not know	Case
Are there previous conclusive reports on this reaction?	+1	0	0	+1
Did the adverse event appear after the suspected drug was given?	+2	-1	0	+2
Did the adverse reaction improve when the drug was discontinued, or a specific antagonist was given?	+1	0	0	+1
Did the adverse reaction appear when the drug was readministered?	+2	-1	0	0
Are there alternative causes that could have caused the reaction?	-1	+2	0	+2
Did the reaction reappear when a placebo was given?	-1	+1	0	0
Was the drug detected in any body fluid in toxic concentrations?	+1	0	0	0
Was the reaction more severe when the dose was increased, or less severe when the dose was decreased?	+1	0	0	0
Did the patient have a similar reaction to the same or similar drugs in any previous exposure?	+1	0	0	0
Total score				6 (Probable)

A review of the drug information for ertapenem available from the USA Food and Drug Administration (FDA) lists that nervous system and psychiatric adverse reactions like seizures, anxiety and insomnia have an occurrence of 0.1-0.5% [13]. Previous reports of ertapenem-induced encephalopathy have more frequently described neurological manifestations such as seizures, asterixis, or gait instability [14-16]. In contrast, our patient presented with florid psychotic symptoms with relatively milder neurological symptoms. Such presentations have been reported as a possible adverse reaction, albeit rare, and with no reliable estimate on their frequency [14].

Renal dysfunction has been identified as one of the important patient-related risk factors for ertapenem encephalopathy [17-19]. Most earlier studies also involve elderly patients or those with multiple comorbidities [17,19,20], whereas our patient is middle-aged and does not suffer from renal impairment. The absence of renal impairment in our case also parallels findings from another report [15] where neurotoxicity occurred despite normal renal function, suggesting that renal dysfunction, while contributory, is not a prerequisite for neurotoxicity. One plausible explanation is the concurrent use of metronidazole, which also acts via GABA antagonism. The additive inhibitory effect on GABAergic transmission could have potentiated the effects of ertapenem on similar pathways. This potential pharmacodynamic interaction between antibiotics acting on similar neural pathways is not well understood. Further studies and pharmacovigilance data could help clarify the mechanisms underlying carbapenem-associated neurotoxicity and identify patient characteristics that confer vulnerability.

One limitation of this report is that causality cannot be definitively established, as other potential contributing factors, such as subclinical inflammatory processes, cannot be fully excluded. Compared with previously reported cases, the onset of symptoms in our patient was slightly delayed (around day 14) and resolution rapid (within three days). Nonetheless, the temporal relationship between ertapanem exposure and symptom onset, along with clinical improvement following withdrawal, provides strong supportive evidence. The short recovery time following drug withdrawal aligns with other reports emphasizing reversibility once the offending agent is stopped [15,18,20]. Collectively, these findings highlight the spectrum of clinical manifestations of carbapenem-induced encephalopathy and emphasize the value of early recognition. Clinicians are encouraged to report similar cases to enhance collective understanding and guide safer prescribing practices. 

## Conclusions

We have presented a case report on ertapenem encephalopathy in a patient with no prior identified risk factors. We hope that this case draws attention to neuropsychiatric complications of ertapenem use, as an important consideration in the differential diagnosis of acute change in mental state. Early recognition and prompt discontinuation of the offending agent can lead to full clinical recovery, reinforcing the importance of careful medication review in acute confusional presentation.
